# Platelet-rich plasma injection vs corticosteroid injection for conservative treatment of rotator cuff lesions: A protocol for systematic review and meta-analysis: Erratum

**DOI:** 10.1097/MD.0000000000025595

**Published:** 2021-04-16

When the article, “Platelet-rich plasma injection vs corticosteroid injection for conservative treatment of rotator cuff lesions: A protocol for systematic review and meta-analysis”^1^, which appeared in Volume 100, Issue 7 of *Medicine*, published, the title appeared incorrectly with “protocol for” in the title. This has since been removed and the title corrected to “Platelet-rich plasma injection vs corticosteroid injection for conservative treatment of rotator cuff lesions: A systematic review and meta-analysis.”
